# Received Cradling Bias During the First Year of Life: A Retrospective Study on Children With Typical and Atypical Development

**DOI:** 10.3389/fpsyt.2020.00091

**Published:** 2020-02-27

**Authors:** Gianluca Malatesta, Daniele Marzoli, Fabio Apicella, Claudia Abiuso, Filippo Muratori, Gillian S. Forrester, Giorgio Vallortigara, Maria Luisa Scattoni, Luca Tommasi

**Affiliations:** ^1^ Department of Psychological, Health and Territorial Sciences, University “G. d'Annunzio” of Chieti-Pescara, Chieti, Italy; ^2^ IRCCS Stella Maris Foundation, Pisa, Italy; ^3^ Department of Clinical and Experimental Medicine, University of Pisa, Pisa, Italy; ^4^ Department of Psychological Sciences, Birkbeck, University of London, London, United Kingdom; ^5^ Centre for Mind/Brain Sciences, University of Trento, Rovereto, Italy; ^6^ Research Coordination and Support Service, Istituto Superiore di Sanità, Rome, Italy

**Keywords:** Autism Spectrum Disorders, infant-holding bias, brain lateralization, retrospective investigation, neurodevelopment, epigenetics, behavioral markers, mother-infant relationship

## Abstract

A population-level left cradling bias exists whereby 60–90% of mothers hold their infants on the left side. This left biased positioning appears to be mutually beneficial to both the mother and the baby's brain organization for processing of socio-emotional stimuli. Previous research connected cradling asymmetries and Autism Spectrum Disorders (ASD), entailing impairment in socio-communicative relationships and characterized by an early hypo-lateralization of brain functions. In this explorative study, we aimed to provide a contribution to the retrospective investigations by looking for early behavioral markers of neurodevelopmental disorders such as ASD. We hypothesized that an atypical trajectory in maternal cradling might be one of the possible signs of an interference in mother-infant socio-emotional communication, and thus of potential neurodevelopmental dysfunctions. To this aim, we examined photos depicting mother-child early cradling interactions by consulting family albums of 27 children later diagnosed with ASD and 63 typically developing children. As regards the first half of the first year of life, no differences were shown between maternal cradling-side preferences in typical and ASD groups, both exhibiting the left-cradling bias in the 0–3 months period, but not in the 3–6 months period. However, our results show dissimilar patterns of cradling preferences during the second half of the first year of life. In particular, the absence of left-cradling shown in typical mothers was not observed in ASD mothers, who exhibited a significant left-cradling bias in the 6–12 months age group. This difference might reflect the fact that mother-infant relationship involving children later diagnosed with ASD might remain “basic” because mothers experience a lack of social activity in such children. Alternatively, it may reflect the overstimulation in which mothers try to engage infants in response to their lack of responsiveness and social initiative. However, further investigations are needed both to distinguish between these two possibilities and to define the role of early typical and reversed cradling experiences on neurodevelopment.

## Introduction

**Figure 1:**
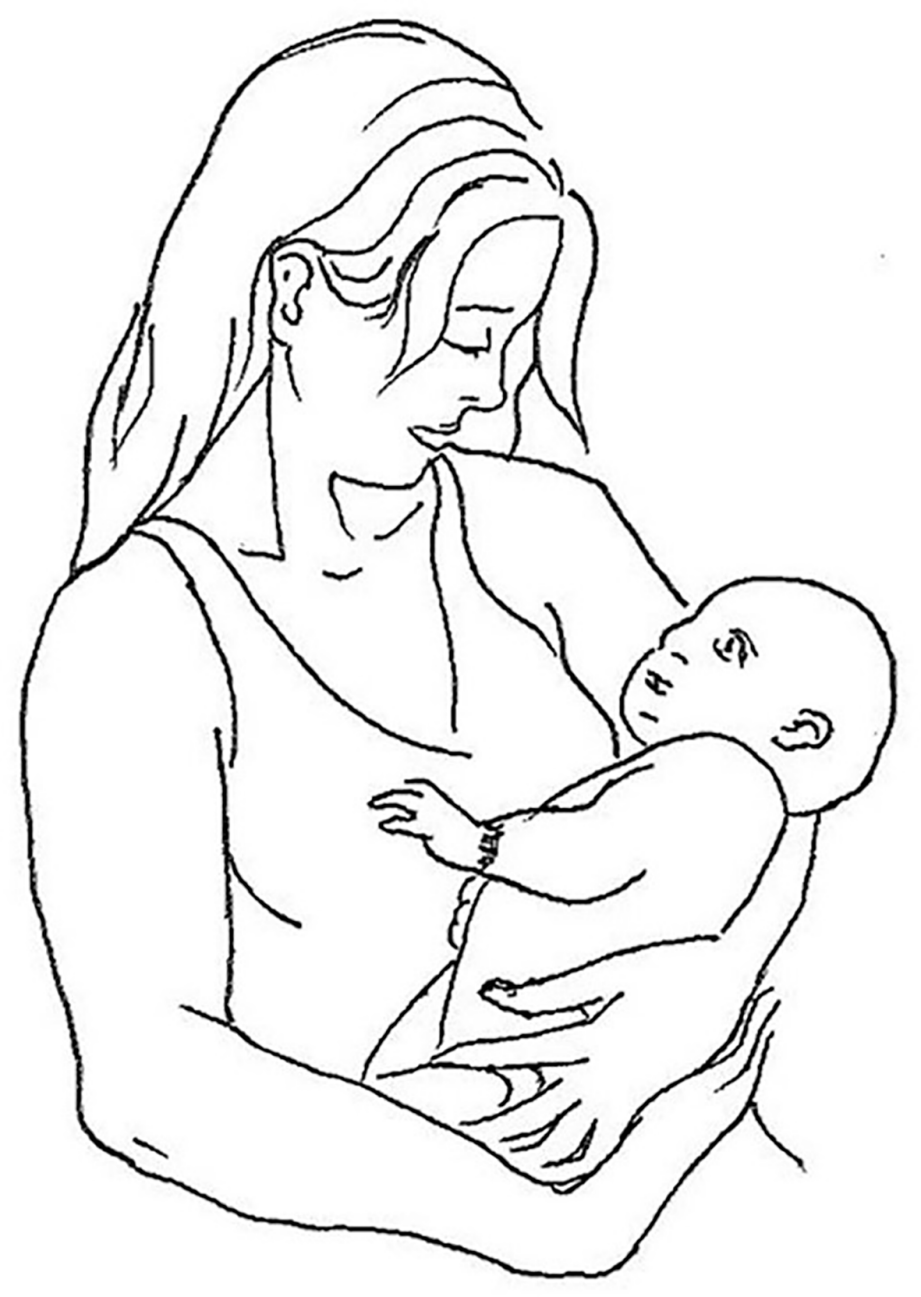
Graphic representation of left-cradling behavior (courtesy of Rocco Cannarsa).

In contrast to right biased motor actions associated with motor sequences and environment-directed behaviors (1, 2), cradling behavior is associated with a bias to the left side of the body whereby an infant is held by an agent (usually the mother) close to her body by using arms and hands (3, 4), as shown in **Figure 1**. Indeed, 60–90% of mothers hold their infants to the left of the vertical midline of their body (5) almost independently of their handedness (6, 7), positioning the head against the chest and/or over the shoulder in their left peripersonal hemispace, and almost always bearing the weight using the left arm. Research shows that the left-cradling bias is strong and fairly stable in the first 18 months of life of the child for mothers. After this period, it was initially shown that left-cradling behavior starts to decline to the point that it is replaced, in some cases, by a right-cradling preference by the time the child is 2 or 3 years old (8). However, in recent longitudinal studies, Scola and colleagues (9) found a slight decrease of left cradling only after 19 months from delivery in mothers, and Todd and Banerjee (10) showed that it was strongest when babies were aged less than 12 weeks.

When a female cradles/holds an infant on the left side, the infant's face is positioned on the left of her visual field and the visual information is processed dominantly by the right hemisphere of the brain, believed to be specialized for the perception and expression of emotion (11, 12). Manning and Chamberlain (13) suggested that, from the mother's point of view, the left-cradling bias facilitates the monitoring of her infant's well-being cues through her left visual and perhaps auditory fields (14) by providing a direct communication projecting to her right hemisphere, specialized for recognizing emotional facial expressions (12, 15). On the other hand, given that many studies showed that newborns are endowed with a predisposition to attend face-like stimuli (16, 17), left-sided cradling would allow the infant to receive the more salient emotional information by means of a constant access to the left side [i.e., the most expressive side (18)] of the mother's face (19).

Besides sleeping and being fed, the newborn life experience is nestled in a close relationship with the adult caregiver (in most cases, the mother), very often expressed in the context of cradling behavior. It would thus be reasonable to propose that cradling is a major framework for most of the neonate's early social and communicative experiences, which provide the epigenetic foundations for the development of later social and communicative abilities (20, 21). In this regard, a growing line of research on behavioral genetics questioned about whether and to what extent changes to the phenotype—especially as regards the occurrence of neurodevelopmental disorders—are under the epigenetic control of imprinting processes not yet fully understood (22).

Using chimeric face tasks, many studies (23–25) have demonstrated that the left-cradling bias is predicted by a typical right-hemispheric specialization in the perception of emotions [see ref (26) for a thorough examination of leftward perceptual and emotional asymmetries]. Therefore, the left bias has been assumed to be associated with better recognition of emotional stimuli presented to the left visual and auditory fields, which are under right-hemispheric control (14). Specifically, Huggenberger and collaborators (27) suggested that cradling side preference is determined by a management of cognitive resources during monitoring emotional signals from the infant face. Vervloed, Hendriks, and van den Eijnde (28) also investigated the effects of the “received” lateral cradling bias, showing that healthy individuals who had been held in the right arm during childhood exhibited in turn a significantly reduced left-bias for emotional faces compared to those who had been held in the left arm. Additionally, Hendriks, van Rijswijk, and Omtzigt (19) suggested that reduced or sub-optimal exposure to face information during infancy (due to a reversed lateral cradling position, i.e., on the right side) might have consequences for the ability to recognize faces and facial expressions later in life. This is likely to occur because the early infant exposure to faces is extremely important not only for fostering the bonding between newborn and caregiver (17), but also for later visual cognitive development (29, 30). Indeed, both male and female observers seem to show an experience-dependent bias of the right hemisphere for the female face, possibly because of the greater incidence of left cradling during the early stages of development, as suggested by refs (31) and (32). Furthermore, studies on non-human vertebrates seem to confirm the presence of an evolutionary right-hemispheric predisposition to process social stimuli to the benefit of an infant's left-sided positioning during interactions with the mother (33) [see ref (34) for a review].

Pileggi and colleagues (35), assuming that the left-cradling bias is fostered by instinctive and right-hemisphere-localized attachment processes that allow individuals to relate to others, found that left-cradling bias is absent in children with Autism Spectrum Disorders (ASD), a population characterized by chronic and severe impairment in empathizing competencies and social relations (36). These findings were corroborated by Fleva and Kahn (37), who showed a negative correlation between left-cradling bias and the presence of autistic traits in adults, and by Malatesta and colleagues, who showed positive correlations between left-cradling bias and both empathy (38) and secure attachment (39). In this regard, it should be pointed out how, compared with typically developing individuals, those with autism are not biased to facial information from the left visual field, as shown by various studies using both eye-tracking and chimeric faces [e.g., see refs (40, 41)]. These studies showed decreased right-hemispheric dominance for emotion processing in this population, different from the patterns of lateralization usually shown by typically developing individuals.

Much evidence has shown that decreased cerebral lateralization is associated with impaired cognitive functions, and it can also emerge behaviorally as mixed handedness [e.g., see ref (42)], given the crucial role that functional asymmetries play during cognitive tasks that require the use of both hemispheres. Hemispheric specialization provides the individuals with several advantages, such as the capacity to exploit in parallel the competences of the left and right hemispheres, to decrease the duplication of execution across hemispheres, and to reduce the initiation of simultaneous and incompatible responses (2, 43). In fact, the existence of a link between glitches in the typical separation of hemispheric functions during brain development and the occurrence of several mental disorders has been hypothesized, as in the case of the communicative shortcomings shown by patients with schizophrenia (44) or other instances of emotion dysregulation disorders in humans and animals (see ref (45) for a review). With regard to this, Forrester and colleagues (46) assessed handedness as a marker of cerebral lateralization in different manual activities both in typical and autistic children, considering that reduced hemispheric specialization in motor behaviors might be an early marker of alterations in brain architecture related to autism onset. Indeed, the study showed that within the context of object manipulation and self-directed behaviors, children diagnosed with autism demonstrated decreased hand dominance compared with their typically developing counterparts. Moreover, Knaus and collaborators (47) showed that ASD is associated with atypical language laterality in adolescents. Specifically, autistic children are characterized by an early hypo-lateralization of brain function compared to typically developing children.

Although Autism Spectrum Disorder (ASD) etiology is still unclear, we now know that such disorders have strong heritable and genetic underpinnings (48) involving 300–500 different genes (49). Remarkably, in their study on relatives, Manning and Denman (50) found that women's left cradling passed down to subsequent daughters and granddaughters, thus revealing genetic influences (through the female line) on lateral cradling tendencies. Along with cradling-side preferences, developmental instability (which in turn has been related to reduced left-cradling tendencies) seems to be passed down from mother—but not father—to children (51), suggesting that genetic and environmental [see also ref (52)] stressors could alter typical cradling asymmetries. Interestingly, a recent study showed that elevated levels of prenatal amniotic oestrogens (which could represent a hormonal stressor) are an important predictor of ASD in boys (53).

To date, data gathered hint at the importance of investigating associations between observations of cradling behavior received by the caregiver and later incidence of ASD, the early detection of which would have crucial implications for therapeutic success of clinical intervention (20, 21). Currently, autism is usually not diagnosed until a child is at least 3 years old, with a mean diagnosis age of 5.7 years (54, 55). Therefore, most recent research used both prospective [e.g., the early observation of newborns “at risk” to develop autism because of previously affected siblings (56)] and retrospective [e.g., analyzing home-movies from the first months of life of autistic children, and their caregivers (57, 58)] methodologies in order to diagnose the condition earlier. These studies indicated that autistic symptoms involve not only social communication and repetitive behaviors, but also influence to some extent motor capacities and the regulation of attention and emotion (59). Analogously, previous findings seem to endorse the opinion that empathy (37, 38), social attachment (35, 39), and emotion lateralization (13, 14) strongly affect early lateral cradling preferences in females. Moreover, a recent study conducted by Forrester and colleagues (60) suggested interesting associations between left-cradling bias and enhanced social processing abilities in (typically developing) 5–6 years old children.

Cradling evidence seems to converge towards a link between reversed cradling behavior, decreased handedness, and atypical development (21). An examination of the cradling bias as a possible early behavioral marker of later typical or atypical development of the child seemed desirable at this point. Thus, we hypothesized that an atypical developmental trajectory in maternal cradling, indicating an interference in socio-emotional communication between mother and infant, might be one candidate epigenetic behavioral marker of ASD in children, arising, and already observable in the first hours after delivery.

We present a retrospective longitudinal study capitalizing on the cradling-side preferences assessed from pictures belonging to family albums. It is rather reasonable to expect that most parents keep a rich collection of images depicting their children since immediately after birth, often including photos depicting the children being cradled. This appeared to be a good proxy for measuring cradling side preference in a sample of mothers of atypically developing children, especially because the retrospective nature of such a survey would reflect the expression of cradling behavior in the months preceding the diagnosis, in the assumption that—*a posteriori*—any behavior could account as a potential marker predicting the later development of the disorder.

The “family photo album” methodology is not new, as witnessed by Manning (61), who examined many photographs from his colleagues' family albums in which they were cradling their infants. He examined photos dividing them according to the age of the cradled child and found that the left-cradling percentage in females was strongest (the figure was between 60 and 70%) when the children were 0-3 months old. In the other age groups (3–6 months, 6–12 months, 1–2 years, > 2 years), females exhibited only a non-significant tendency to cradle on the left, the left-cradling bias decreasing after the third month after child birth. These findings are consistent with Todd and Benerjee's (10) recent reports.

## Methods

### Participants

Mothers (age range at the time of evaluation: 29–50; M = 40.52; SD = 5.05) of 63 typical children (age range at the time of evaluation: 1.4–16 years; M = 8.44; SD = 3.41) and mothers (age range at the time of evaluation: 27–55; M = 38.59; SD = 6.12) of 27 children diagnosed with ASD (age range at the time of evaluation: 1.9–16 years; M = 4.78; SD = 3.43) took part in the study. Mothers in the typical group were recruited from pediatrics practices and primary and secondary schools of Italian regions Molise, Abruzzo and Marche. Participants in the atypical group were recruited from all over the country among parents whose children had been diagnosed with ASD at “Stella Maris IRCCS” of Pisa (Italy). Only participants with a certified diagnosis of ASD according to medical certification were recruited in the atypical group. All mothers participating in the study provided written informed consent to participate in the study by signing an authorization form. Neither invasive nor risky procedures were involved, and the data were analyzed anonymously. The study was carried out in accordance with the principles of the Declaration of Helsinki and following the approval of the Italian “National Institute of Health” (“Istituto Superiore di Sanità”) ethical committee (Ethical Committee Approval Number: PRE 469/16).

### Procedure

Mothers of children were approached by the experimenter under the supervision of psychologist/doctor/teacher, depending on the context in which they were recruited: schools or pediatrics practices in the case of the typical/control group; in the waiting rooms of “Stella Maris IRCCS” in the case of the atypical/experimental group.

Once recruited, mothers were asked to fill in a take-home survey concerning their child in which they were required to indicate preliminary information about both the child (sex; diagnosis; birth order; handedness) and themselves (age; handedness). Then, participants were asked to consult their family photo albums, specifically seeking photographs in which mothers were cradling their children, and to make a single entry on a first grid, for photos in which the child was under 12 months of age, or on a second grid, for photos in which the child was over 12 months of age. Using the baby's head as a reference point, participants were required to indicate the side on which the child was being held in each photo, taking note of the age (in years and months) of the baby at the time of capture.

## Results

We collected data from 1,667 photos (range per participant: 3–101; M = 26.46; SD = 20.86) in which mothers were cradling their typical children (N = 63; control group) and 543 photos (range per participant: 0-51; M = 20.11; SD = 13.08) in which mothers were cradling their children later diagnosed with ASD (N = 27; experimental group). Two mothers belonging to the atypical group did not provide any photos in which they were cradling their children.

In order to trace a cradling trajectory both in typical and in atypical development of children, we carried out an analysis splitting age groups on the basis of Manning's (61) photo-categories. We examined the following categories of photos collected per age group of the child: 0–3 months; 3–6 months; 6–12 months; 1–2 years. **Table 1** shows the distribution of photos in each age group:

**Table 1:** Number of collected photos depicting mothers cradling their typical (control group) and atypical (ASD; experimental group) per age group of the child.

Child development [N]	0–3 months (mean; SD)	3–6 months (mean; SD)	6–12 months (mean; SD)	1–2 years (mean; SD)
Typical [62]	**390** (6.19; 5.63)	**262** (4.19; 5.03)	**336** (5.33; 4.85)	**380** (6.03; 6.75)
Atypical (ASD) [27]	**166** (6.15; 6.26)	**67** (2.48; 2.46)	**119** (4.41; 5.03)	**139** (5.15; 6.68)

Within each age group, only participants who provided at least 4 maternal cradling photos were included in the data analysis. Then, a cradling laterality quotient (CLQ) was computed for each participant as $\frac{right\;photos-left\;photos}{right\;photos\;+\;left\;photos}$ with participants scoring from -1 (all left photos) to +1 (all right photos). Data were analyzed with SPSS Statistics Version 20 (Armonk, NY, USA).

### Age Group 0–3 Months

Thirty-seven participants of the typical group and 18 participants of the atypical group provided at least 4 maternal cradling photos in which infants were aged 0–3 months. The CLQ of mothers of typical children significantly differed from 0, showing a left-cradling bias (N = 37; M = -0.231 [61.55% of left cradling]; SD = 0.616; *t_(36)_* = -2.287; *p* = 0.028; *d =* -0.376; CI = -0.437, -0.26), and a similar pattern (albeit not significant) was observed for mothers of ASD children (N = 18; M = -0.208 [60.42% of left cradling]; SD = 0.442; *t_(17)_* = -2.002; *p* = 0.062; CI = -0.428, 0.011). Lateral cradling preferences in mothers of typical and ASD children did not differ significantly (*t_(53)_* = -0.143; *p* = 0.887).

### Age Group 3–6 Months

Twenty-four participants of the typical group and seven participants of the atypical group provided at least four maternal cradling photos in which infants were aged 3–6 months. The CLQ of mothers of typical children significantly differed from 0, showing a right-cradling bias (N = 24; M = 0.245 [37.75% of left cradling]; SD = 0.573; *t_(23)_* = 2.099; *p* = 0.047; *d* = 0.428; CI = 0.004, 0.487), and a similar pattern (albeit not significant) was observed for mothers of ASD children (N = 7; M = 0.195 [40.25% of left cradling]; SD = 0.553; *t_(6)_* = 0.930; *p* = 0.388; CI = -0.317, 0.706). Also in this case, lateral cradling preferences in mothers of typical and ASD children did not differ from one another (*t_(29)_* = -0.208; *p* = 0.837).

### Age Group 6–12 Months

Thirty-five participants of the typical group and 14 participants of the atypical group provided at least four maternal cradling photos in which infants were aged 6–12 months. The CLQ of mothers of typical children did not differ from 0, showing a slight and no significant left-cradling bias (N = 35; M = -0.059 [52.95% of left cradling]; SD = 0.679; *t_(34)_* = -0.514; *p* = 0.61; CI = -0.292, 0.174); in contrast, mothers of ASD children showed a strong left-cradling bias (N = 14; M = 0.426 [71.29% of left cradling]; SD = 0.543; *t_(13)_* = -2.933; *p* = 0.012; *d* = -0.67; CI = -0.740, -0.112). Although the control and the experimental group showed a different pattern, this difference did not reach statistical significance (*t_(47)_* = -1.801; *p* = 0.078).

### Age Group 1–2 Years

Thirty-four participants of the typical group and 12 participants of the atypical group provided at least four maternal cradling photos in which infants were aged 1–2 years (i.e., between the 12^th^ and the 24^th^ month of child's age). Both the CLQ of mothers of typical children (N = 34; M = -0.061 [53.05% of left cradling]; SD = 0.602 *t_(33)_* = 0.588; *p* = 0.561: CI = -0.150, 0.271) and that of mothers of ASD children (N = 12; M = 0.073 [53.65% of left cradling]; SD = 0.589; *t_(11)_* = 0.431; *p* = 0.675; CI = -0.301, 0.448) did not differ from 0, showing no lateral cradling preference for this age group. Moreover, no difference was observed between the control and the experimental group (*t_(44)_* = 0.063; *p* = 0.95).

**Figure 2:**
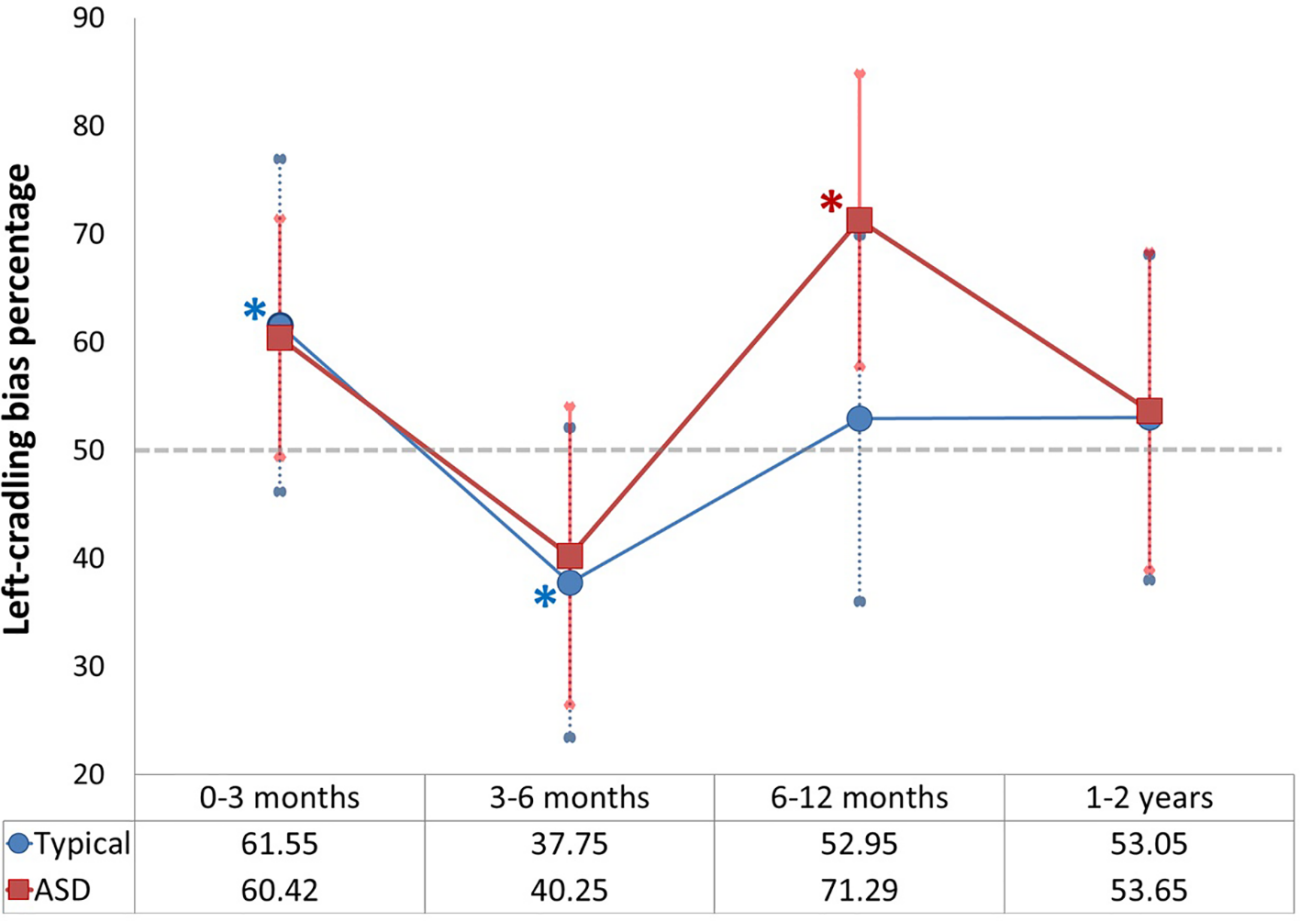
Left-cradling percentage based on cradling laterality quotient (CLQ) of mothers of typical and Autism Spectrum Disorders (ASD) children in all age groups (the asterisks indicate significance of p < 0.05 [in blue as regards typical children; in red as regards ASD children]; the grey dashed line indicates the chance level [50%]; error bars indicate standard deviations).

**Figure 2** depicts the mixed cross-sectional longitudinal trajectory of received maternal left cradling in the first two years of life of both groups of children.

## Discussion

The aim of this explorative study was to describe a retrospective longitudinal trajectory of maternal cradling side preference for children diagnosed with ASD—compared with that of typically developing children—in the first two years of life. Since it is impossible, at present, to observe autistic children before the second year of life (due to age of diagnosis), we decided to carry out an “indirect retrospective observation” of mothers using family photos in which they were cradling their children. Mothers were required to provide the age of the child for each photo in order to depict the longitudinal temporal cradling trajectory, according to the age groups used by Manning (61).

No difference was found in lateral cradling preferences between the mothers of typical and autistic children in the first three months after delivery, that is the period in which left-cradling bias is particularly strong in healthy mothers (3, 4, 10) but not in mothers with affective symptoms such as stress, anxiety or depression (38, 62, 63). The left-cradling bias was clearly apparent from photos of the first age group (0–3 months) in both groups: significantly in typical children and trending towards significance in ASD children (probably due to the smaller sample size). In this regard, it is important to note that the photo laterality quotient is an index not coming from a direct observation, and is thus susceptible to many potential factors that might intervene on the bias detection. Indeed, photos can capture a given moment, but they might not be systematically indicative of the actual cradling behavior involving mother and child. However, scoring the family photo albums was successfully used by Manning (61), and also in the present study a left-cradling bias (61.55%) was observed in the first three months, which confirms the usefulness of this method to obtain information not accessible otherwise.

As shown by Manning (61) and, more recently, by Todd and Banerjee (10), after the third month of life of the child there is a remarkable decline of the left-cradling preference in mothers. The present data replicated such a decline from the 12^th^ week, and also indicated a clear right-cradling bias observable in mothers of typical children in the 3–6 months age group. This right bias was also present in mothers of ASD children, albeit it was not significant. In this regard, it should be noted that only seven participants of the ASD group provided an acceptable number of maternal cradling photos for this age group, thus making this comparison the least reliable of the study.

Interestingly, in the second half of the first year of life (age group: 6–12 months), mothers of children with autism exhibited a strong and significant increase of left-cradling bias, whereas the mothers of typical children did not show any lateral preference. In the subsequent age group (1–2 years), data did not show any difference between groups.

In this respect, it should be noted how past research suggested that cradling lateral preferences might not be due exclusively to the right-hemispheric specialization for emotion processing (6, 64). Indeed, a significant relationship between hemispheric lateralization and cradling-side bias is observed only for “basic” holding relationships, in particular those in which the held or cradled element (e.g., a doll) does not provide a feedback in response to the holding side or position. On the other hand, “advanced” holding relationships are characterized by a considerable involvement between the cradling and cradled individuals (e.g., a mother with her infant) (6, 64). In this case, the mother could gradually adjust her lateral preference in response to the infant's activity, and there might be more room for the effect of affective or psychological factors [e.g., insecure attachment, lack of empathy, depression (38, 39)]. Thus, it could be speculated that mother-infant relationships involving children later diagnosed with ASD might remain “basic” because mothers experience a lack of social activity in such children. Actually, many retrospective and prospective studies have reported that infants later diagnosed with autism have social difficulties in reciprocal interactions with their caregiver that were present since the first months of life (65). Muratori and colleagues (66) showed that infants later diagnosed with autism, compared with children with typical development, exhibited significantly worse performance in tasks that required the ability to shift attention from non-social to social stimuli, e.g., the orienting-to-name ability that usually increases around the 9^th^ month (67). The lack of socially motivated engagement becomes an early specific signal of autism by 12 months of age of child, with respect to other neurodevelopmental disorders (57). Furthermore, Dundas, Gastgeb and Strauss (68) showed a left bias for faces in typical children arising around 11 months, whereas children with high risk of autism did not show such a bias (69). Similarly, Jones and Klin (70) found that ASD children showed a developmental decline in eye fixation from about 2 until 24 months of age, despite appearing to begin at normative levels prior to this drop.

Parents of children later diagnosed with autism seem to perceive, long before diagnosis, the lack of responsiveness and social initiative of their infants. Indeed, they engage themselves increasingly more in a close relationship and stimulate their children more than parents of neurotypical children (71). Many investigations reported that mother-child relationships involving ASD children showed qualitative differences with respect to those involving typically developed children (72). Mothers of autistic children, actually, tend to engage more in physical contact with their infants and perform more high-intensity child-directed behaviors (73). In general, compared with parents of typical children, parents of autistic children show more positive strategies of parenting style, probably in order to improve the attachment with their children (74). This over-responsive engagement style may represent a reaction, implemented precisely in the second semester by parents, to the atypical development exhibited by ASD infants (75).

Such evidence seems to suggest that the significant increasing of the left-cradling bias we observed in mothers of ASD children (during the 6–12 months period) might be an unconscious outcome of the attempts carried out by parents, and especially by the mother, to recover their infants to a more vivid emotional activity. A body of work, indeed, indicates that the defining features of autism are not present at the first 6 months of age but begin to emerge later (76). For example, a decreasing vocalization and an increasing of non-social babbling (77) and more frequent and longer repetitive movements (78) have been described as characterizing this period.

The present results corroborate the idea that left cradling might be considered as an early marker of the quality of the search for emotional closeness between the cradling and cradled individuals (or at least, in the present case, of the parents' efforts to improve such a “basic” relationship).

Although possible stressing factors linked to the mother seem to be involved in both ASD onset (53) and reduced left-cradling preferences (51, 52), the fact that these variables were not related in the present study suggests that they result from different causes.

Finally, although our findings should be considered as preliminary, above all because of the small sample, the results reported here might encourage further studies aimed at investigating whether atypical patterns of cradling-side preferences in children with ASD might reflect either: (i) differences in the nature of the mother-infant relationship (“basic” or “advanced”) or (ii) the indirect overstimulation in which mothers try to engage infants in response to their lack of responsiveness and social initiative, and (iii) whether they can be used as a non-invasive behavioral marker for the earlier identification (already in the first year of the infant's life) of children at risk of ASD.

## Data Availability Statement

All datasets generated for this study are included in the article/**Supplementary Material**.

## Ethics Statement

All participants provided written informed consent to participate in the study by signing an authorization form. Neither invasive nor risky procedures were involved, and the data were analyzed anonymously. The study was carried out in accordance with the principles of the Declaration of Helsinki and following the approval of the Italian “National Institute of Health” (“Istituto Superiore di Sanità”) ethical committee (Ethical Committee Approval Number: PRE 469/16).

## Author Contributions

GM, LT, DM, and MS conceived and created the experiment. GM, CA, FA, and FM conducted the experiment. LT, MS, FM, GF, FA, and GV supervised all the phases of the study. GM, DM, LT, and MS analyzed the results. GM wrote the paper. All authors reviewed the manuscript.469/16).

## Funding

This project has been partially supported by the Fondazione Italiana Autismo Onlus (Project W17, 'Italian Network for the early detection of ASD') and the “Italian Autism Spectrum Disorders Network: Filling the gaps in the National Health System care” NET-2013-02355263.

## Conflict of Interest

The authors declare that the research was conducted in the absence of any commercial or financial relationships that could be construed as a potential conflict of interest.
